# Role of CPEB3 protein in learning and memory: new insights from synaptic plasticity

**DOI:** 10.18632/aging.103404

**Published:** 2020-07-02

**Authors:** Wen Rui Qu, Qi Han Sun, Qian Qian Liu, Hong Juan Jin, Ran Ji Cui, Wei Yang, De Biao Song, Bing Jin Li

**Affiliations:** 1 Department of Hand Surgery, The Second Hospital of Jilin University, Changchun, Jilin Province, China; 2 Jilin Provincial Key Laboratory on Molecular and Chemical Genetic, The Second Hospital of Jilin University, Changchun, China; 3 School of Pharmacy, Jilin University, Changchun, China; 4 Department of Plastic and Reconstructive Surgery, The First Hospital of Jilin University, Changchun, China

**Keywords:** CPEB3, synaptic plasticity, RNA-binding proteins, long term memory, plasticity-related proteins

## Abstract

The cytoplasmic polyadenylation element-binding (CPEB) protein family have demonstrated a crucial role for establishing synaptic plasticity and memory in model organisms. In this review, we outline evidence for CPEB3 as a crucial regulator of learning and memory, citing evidence from behavioral, electrophysiological and morphological studies. Subsequently, the regulatory role of CPEB3 is addressed in the context of the plasticity-related proteins, including AMPA and NMDA receptor subunits, actin, and the synaptic scaffolding protein PSD95. Finally, we delve into some of the more well-studied molecular mechanisms that guide the functionality of this dynamic regulator both during synaptic stimulation and in its basal state, including a variety of upstream regulators, post-translational modifications, and important structural domains that confer the unique properties of CPEB3. Collectively, this review offers a comprehensive view of the regulatory layers that allow a pathway for CPEB3’s maintenance of translational control that guides the necessary protein changes required for the establishment and maintenance of lasting synaptic plasticity and ultimately, long term learning and memory.

## INTRODUCTION

Aberrant synaptic plasticity has been implicated in many neurodegenerative and neuropsychiatric diseases including Parkinson’s disease, Huntington’s disease, Alzheimer’s disease, and schizophrenia [1–4]. Synaptic plasticity is the biological process by which neural synapses strengthen or weaken their response to incoming stimuli over time. This process forms the basis of long-term learning and memory where lasting increase in synaptic strength is termed long-term potentiation (LTP) and lasting decreases termed long-term depression (LTD) [5–7]. The molecular basis of LTP/LTD rests in the on-going morphological and biochemical alterations at the synaptic junctions that modulate the response to incoming stimuli. Proteomic changes are a major component of this neuromodulation, particularly, the interaction between RNA-binding proteins and their targets [8–11]. Perhaps none are more impactful than the regulation of glutamatergic receptor elements such as N-methyl-D aspartate (NMDA) or α-amino-3-hydroxy-5-methyl-4-isoxazolepropionic acid (AMPA) receptor subunits, which modulate neural excitability and shape cell to cell communication and subsequently, establish plasticity. Though ample regulatory mechanisms exist to drive synaptic plasticity, in this review, we highlight cytoplasmic polyadenylation element-binding protein 3 (CPEB3) and its role in the regulation of plasticity-related protein regulation. We describe its role in learning and memory as well as delve into the molecular studies that have elucidated its dual role as a molecular regulator of translation and to some extent transcription, extrapolating how CPEB3 may contribute to the overall achievement of lasting neuromodulation within the synaptic junction.

## CPEBs in the brain

Synaptic plasticity forms the basis of learning and memory capacity through its lasting modulation of neuronal excitability [12, 13]. Repeated or continuous synaptic activation can result in modification of existing synaptic proteins through either direct or second messenger effects, initiating protein synthesis changes in the neuron, structurally altering the synapse and contributing to long-term changes in synaptic strength [14, 15]. Alterations to the mechanisms which allow this modulation contribute significantly to neurological diseases from neurodegenerative disorders (Alzheimer’s, Parkinson’s, and Huntington’s disease) to neuropsychiatric diseases such as depression and schizophrenia [16–19]. Indeed, changes to components regulating or rendering synaptic plasticity are often some of the earliest signs of these diseases.

**Figure 1:**
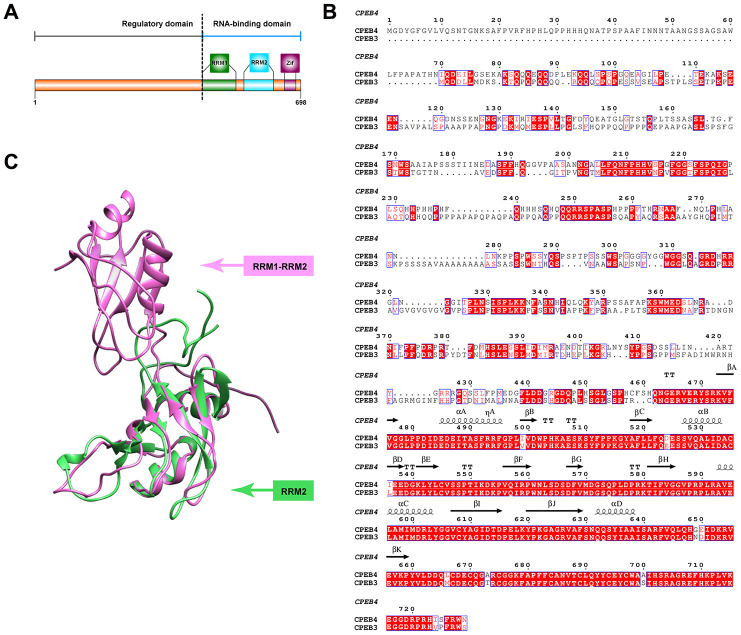
**Structural features of CPEB3.** (**A**) Primary features of CPEB3. The C terminal fragment of CPEB3 contains two highly conserved RNA recognition motifs (RRM1in forest green and RRM2 in blue) and one zinc-finger domain (Zif, in purple). A highly variable regulatory domain is embedded in the N terminal part of CPEB3. (**B**) Sequence alignment of human CPEB3 and CPEB4. Highly conserved residues between CPEB3 and CPEB4 are marked in red. The secondary structural element labeled on top of the corresponding residues are based on the reported fragment structure of CPEB4 (PDB number: 2MKJ). Numbering of amino acids corresponds to the CPEB4 protein. (**C**) Superposition of the reported CPEB3’s RRM1(PDB number:2RUG) and CPEB4’s RRM1 and RRM2 (PDB number: 2MKJ). The structure of CPEB3’s RRM1 and CPEB4’s RRM1 and RRM2 are displayed in cartoon and colored with forest green and pink, respectively.

Many of these disease-related alterations occur through the interaction of RNA-binding proteins which are essential for the control of spatial-temporal plasticity-related protein (PRP) production [20–24]. Consequently, these changes impact the regulation of the transport, translation and/or stability of PRP RNA. Among translational regulators, the cytoplasmic polyadenylation element-binding protein (CPEB) family is a key RNA-binding protein family, one that plays a role in modulating the strength of glutamatergic synapses through the translational regulation of several PRP RNAs in neurons [25–30]. This RNA-binding protein family controls cytoplasmatic polyadenylation and translation of target mRNAs at synapses via a self-perpetuating, functional prion-like conformations [31, 32]. This prion-like process can shift CPEB subtypes from monomers into alternative, self-propagating conformations capable of aggregating [33–38]. In vertebrates, 4 members of the CPEB family (CPEB1 (referred to commonly in early literature as CPEB), CPEB2, CPEB3, and CPEB4) have been identified, all of which are highly expressed in the nervous system including the hippocampus, olfactory bulb, cerebellum, and peripheral afferent sensory neurons [36, 39, 40]. While all CPEBs play a role in translational regulation, CPEB1 differs in its recognition and regulatory elements. The earliest CPEB (known now as CPEB1) was discovered and characterized in Xenopus oocytes. CPEB1 achieves translation of mRNA via recognition of cytoplasmic polyadenylation elements (CPE) in target mRNA (regulated by Maskin) and subsequent binding of the cap-binding factor eIF4E (Huang et al 2006). Unlike CPEB1, CPEB2-4 differ in their regulation of target mRNA. While CPEB2-4 share RNA binding elements with CPEB1, they differ in their regulatory domains and expression patterns. Particularly, CPEB3-4 have distinct U-rich loop motifs suggesting targets unique to those subgroups (Fernandez-Miranda et al 2012). In general, all CPEBs have a carboxy terminal region composed of two RNA recognition motifs (RRMs) and two zinc finger-like motifs complemented by a highly variable N-terminal (Figure 1). Finally, there are highly conserved residues between the CPEB3 and CPEB4 protein which could hint at CPEB3’s secondary structure/function (Figure 1B), of which not much is currently known. It has been reported that the RNA binding domain of CPEB3-4 is capable of recognizing secondary structure of RNA and that the zinc finger domain is required for stable RNA binding while both the RRM1 and RRM2 are required for binding specificity (Huang et al 2006).

## Role of CPEB3 in learning and memory

While CPEBs play a wide range of biological roles, in the brain, CPEB activity is generally modulated by external signals that make them effective synapse-specific protein stabilizers [41–43]. Early work in Aplysia neurons showed that blocking Aplysia CPEB (ApCPEB, non-mammalian homolog of CPEB3) at the synapse prevented the stable maintenance of long-term facilitation (LTF) [34, 44–47]. In Drosophila, the CPEB Orb2 (ortholog of ApCPEB) demonstrates a role in synaptic plasticity and is required for long-term conditioning of male courtship behavior [48–54].

At its core, CPEB3 is involved in synaptic protein regulation which is necessary for the maintenance (but not acquisition) of memory. By acting as a negative regulator of targets like AMPA receptor subunits GluA1 and GluA2 CPEB3 maintains long term synaptic plasticity [35]. In mice, early evidence of CPEB3’s role in synaptic plasticity and memory came from the observed elevation of CPEB3 mRNA in the hippocampus after kainate-induced seizure, indicating it is an immediate early gene product of synaptic activation and likely plays a role in modulating neuronal function [36]. Evidence that CPEB3 may be involved in lasting modulation comes from its increased presence in the synapse, which occurs 30 minutes after glutamate or glycine-induced LTP in hippocampal cultures (Fiorti et al 2015). Indeed, the persistence of long term memory was found to be specifically impaired by knockdown of CPEB3 after memory consolidation in one study [55]. Long term memory maintenance was also ascribed to CPEB3 activation and consequent interaction with actin in another animal study (Stephan et al 2015). A report by Chao et al 2013 further substantiates the negative regulatory role of CPEB3 in learning and memory as knockout mice demonstrated enhanced spatial memory preceded by elevated glutamate signaling and enlargement of spine morphology in excitatory pyramidal neurons (Chao et al 2013). Overall, the functional role of CPEB3 appears to contrast the role of CPEB1 which has been characterized as the extinction of hippocampus dependent long term memory [56] and the quiescence of short-term contextual (fear) memory [25]. This perhaps indicates that CPEB1 and CPEB3 are complimentary in their functional capacity to modulate short-term contextual memory and long-term memory, respectively. In humans, CPEB3 is co-localized in the hippocampus with the synaptic protein synaptophysin (Huang et al 2006). In a 2009 study, a single nucleotide polymorphism in the highly conserved intronic sequence of the human CPEB3 gene was found to be associated with the decreased ability to perform verbal episodic memory tasks in humans [57]. Collectively, these findings suggest a functional role for CPEB3 in both short term and long term memory performance. It should be noted that CPEB3 not only plays an important role in LTP but in LTD as well, as CPEB3 knockout neurons demonstrate impaired evoked LTD (important for diminishing certain types of memory), reduced spine width, and reduced pools of synaptic proteins [58].

## Plasticity related proteins and CPEB3

The synaptic plasticity which confers learning and memory is possible due to the molecular mechanisms that allow for swift and lasting, stimuli-dependent modulation of the proteins that play direct roles in synaptic transmission [59–62]. Neurons generally increase synaptic PRPs through the capture and/or local translation of molecules trafficked from the soma to the synapses [63–65]. Additionally, PRPs can be produced through the activation of pre-existing dormant mRNAs at the dendrites [27, 66, 67]. RNA-binding proteins such as CPEB3 play a critical role in these types of PRP regulation, shaping their abundance and localization to drive synaptic plasticity.

CPEB3 regulates the translation of several PRP RNAs, including NMDA receptor subunit 1 (NR1), AMPA-type glutamate receptor subunits glutamate A2 (GluA2) and glutamate A1 (GluA1), postsynaptic density protein 95 (PSD95), and the cytoskeletal protein actin, and all of which have significant roles in synaptic plasticity (Table 1) [68–71]. CPEB3 loss of function studies in mice have predominantly been used to demonstrate the role of CPEB3 as a negative regulatory of these and other PRPs which all play a role in tapering the strength of the synapse in a memory-related context. These studies also highlight parallel roles for CPEB3 in modulating the electrophysiology and structural properties of local synapses [25].

**Table 1:** CPEB3 targets that play a role in synaptic plasticity.

	**Synaptic role**	**Effect of CPEB3**	**References**
**GluA1**	regulate dendritic spines and functional synases	negative translational regulation by CPEB3 that is reversed by Neurl1-induced ubiquitination of CPEB3	Paupolous et al 2011
**GluA2**	formation of functional AMPARs	translational repression by interaction with SUMOylated CPEB3 via the RRM1 domain; neural stimulation induced translational increase	Hunag et al 2006; Ford et al 2019; Stephan et al 2015
**NR1**	regulate surface expression of NMDARs	translational repression by CPEB3 via spatial sequestration	Chao et al 2013
**NR2A**	regulate surface expression of NMDARs	translational repression by CPEB3 via spatial sequestration	Chao et al 2013
**NR2B**	regulate surface expression of NMDARs	translational repression by CPEB3 via spatial sequestration	Chao et al 2013
**actin**	facilitates synaptic protein transport	CPEB3-mediated translational reperssion; neural stimuation-induced translational increase	Stephan et al 2015
**PSD95**	regulate synaptic strength	direct binding and translational repression by CPEB3	Chao et al 2013

GluA1: glutamate A1; GluA2: glutamate A2; NR1: NMDA receptor 1; NR2A=NMDA receptor 2A; NR2B: NMDA receptor 2B; PSD95: postsynaptic density protein 95.

**Figure 2:**
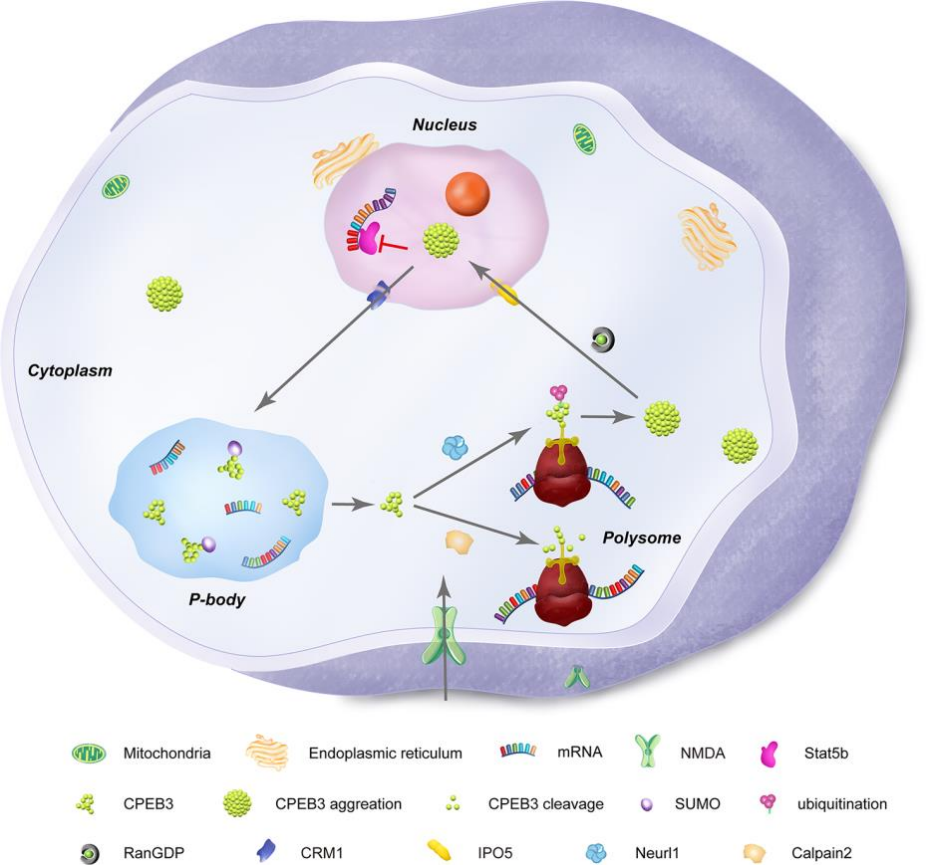
**Subcellular distribution and functional states of neuronal CPEB3.** CPEB3 is expressed both in nuclear and cytoplasmic fractions. In the nucleus, CPEB3 is synthesized and exported to the cytoplasm by a CRM1-mediated nuclear export signal. In the cytoplasm, CPEB3 plays a dual role in translation of target mRNA, largely influenced by the glutamatergic activation of the local synapse. In its basal state, CPEB3 is soluble, SUMOylated, and monomeric. This form of CPEB3 recruits target mRNA into P-bodies. NMDA stimulation signals transformation of CPEB3 by 1) calpain 2-mediated cleavage of CPEB3 interacting domains that promote its monomeric state or 2) Neurl1-mediated ubiquitination that promotes CPEB3 aggregation. CPEB3 aggregation promotes a functional switch into a translational activation state, generally occurring in polysomes. Finally, cytosolic CPEB3 can be translocated back into the nucleus by the importin IPO5 (Ran GDP-dependent) in a CMR1–mediated manner, where CPEB3 then interacts with Satb5 to repress transcription of Satb5-interacting mRNA.

In its basal state, CPEB3 represses the translation of GluA1 and GluA2 in hippocampal neurons (*in vitro*), as confirmed by the upregulation of the AMPA subunits in the hippocampus of conditional CPEB3 knockout mice [55]. Interestingly, stimuli-dependent increases in the AMPA subunits have been experimentally shown to be regulated by CPEB3-driven increases in translation of the targets through polyadenylation, suggesting a functional switch of the RNA-binding protein occurs in the context of synaptic stimulation [55] (Figure 2).

In addition to AMPARs, CPEB3 knockout experiments have shown that NMDAR subunits such as NMDA receptor 1 (NR1), NMDA receptor 2A (NR2A) and NMDA receptor 2B (NR2B) as well as the postsynaptic density protein 95 (PSD95) are translationally up-regulated, suggesting a repressive role of CPEB3 [25, 58]. Similarly, in response to induced c-LTD, degradation of GluA1 and PSD95 are notably diminished in CPEB3 knockouts but rescued to some degree by exogenous expression of CPEB3. This indicates that CPEB3 plays an important role in activity-dependent reduction of synapse efficacy. After NMDA stimulation, CPEB3 is initially accumulated in the nucleus [26] but significantly degraded after 2 hours [27], inducing de-repression of PRPs.

**Figure 3:**
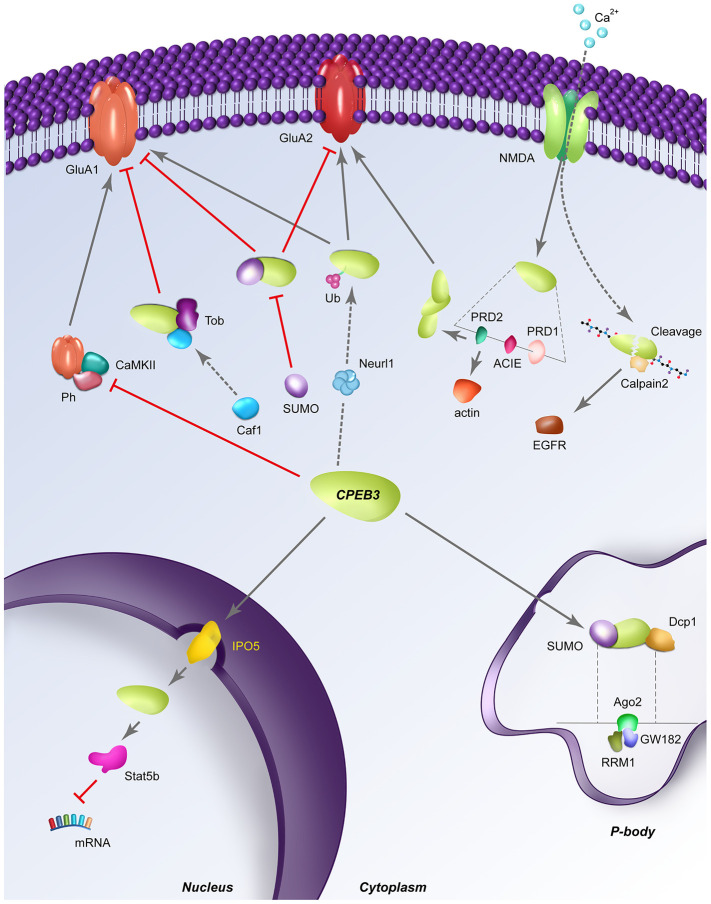
**Dynamic regulatory pathways of CPEB3.** Red perpendicular lines represent an inhibitory relationship; Arrows indicate a stimulatory relationship; SUMO: sumoylation; Ub: ubiquitination; Ph: phosphorylation; RRM1: RNA recognition motif 1; dashed lines indicate a structural expansion of a portion of the CPEB3 gene is located below. (**A**) CPEB3 inhibits the NMDA-dependent phosphorylation by CAMKII, allowing the expression of CPEB3 targets in a stimulation-dependent manner. (**B**) In its basal state, Tob binds CPEB3, recruiting the deadenylase Caf1 for the deadenylation of CPEB3 targets like GluA1. (**C**) SUMOylated CPEB3 (*depicted in the cytoplasm*) inhibits the expression of downstream targets GluA1 and GluA2; Sumoylated CPEB3 also downregulates further CPEB3 sumoylation in a negative feedback loop. SUMOylated CPEB3 (*depicted in P body*) is associated with the P-body protein mRNA de-capping enzyme 1 (Dcp1); sequestration of CPEB3 to P-bodies is specifically driven by the interaction of the RRM1 domain of CPEB3 to Ago2 and GW182 (D) Neurl1 induces CPEB3 monoubiquitination, inversing the basal repressive role of CPEB3 on targets like GluA2. (**E**) NMDA stimulation induces CPEB3 activation translation of actin through direct binding of the actin cytoskeleton interaction element (ACIE). The PRD1 domain flanking the ACIE promotes aggregation of CPEB3s to one another, further promoting the translation of other CPEB3 targets like GluA2. (F) Stimulation-induced calcium influx triggers the cleavage of CPEB3 by calpain 2, leading to proteolysis of the N-terminal repression motif to activate translation of CPEB3 targets like EGFR. (**G**) Nuclear translocation of CPEB3 from the cytoplasm occurs through the karyopherins IPO5, after which CPEB3 associates with the transcription factor Stat5b to downregulate transcription of Stat5b targets like EGFR.

NMDA receptor (NMDAR)-dependent degradation of CPEB3 was found to occur through cleavage by calpain 1 and calpain 2, though only calpain 2 deficiency was associated with impaired proteolysis of CPEB3 [27]. Elevated calcium influx resulting from NMDA stimuli activates calpain 2 and cleaves CPEB3 at the N-terminal repression motif (but not the C-terminal RNA binding domain) to achieve non-polyadenylation activation of CPEB3-RNA targets. Moreover, the degradation of CPEB3 is necessary for the translational upregulation of EGFR as demonstrated in CPEB3 knockdown neurons. Epidermal growth factor receptors (EFGRs) are critical for normal astrocyte development and neuronal survival (Figure 3). Incidentally, epidermal growth factor (EGF) has been shown to play a role in enhancing LTP in the hippocampus, suggesting a function as a neuronal modulator. EGFR has also been pharmacologically shown to impact spatial learning and memory in mice [26]. Interestingly, the receptor tyrosine kinase EGFR is a target gene transcriptionally activated by Stat5b, which is downregulated by CPEB3 in the nucleus. In CPEB3 knockdown neurons, EGFR expression is increased and when stimulated with EGF, alters the kinetics of downstream signaling. Together, this indicates that CPEB3 plays a novel role in the nucleus to suppress Stat5b-dependent EGFR transcription, consequently negatively regulating EGFR signaling in neurons [72].

CPEB3 have also shown co-localization with actin in hippocampal neurons and synaptosomes [73]. Further experimentation in yeast found that disruption of actin filamentation inhibited the aggregation of CPEB3. Indeed, CPEB3 binds actin mRNA in hippocampal cells and demonstrates an inhibitory effect on actin translation *in vitro*. Similar to NMDA, stimulation alters the repressive role of basal CPEB3 on actin, increasing actin in the context of CPEB3 prion formation via the prion-like domain 1 (PRD1). Through deletion experiments in yeast, this domain was found to possess two Q/N-rich, aggregation promoting domains surrounding a regulatory element with confirmed binding capacity between CPEB3 and the actin cytoskeleton [73]. These CPEB3 and plasticity related protein interactions are illustrated in Figure 3.

## Molecular determinants of CPEB3 function

### Stimulation-dependent regulation of CPEB3

### CPEB3 aggregation

In the basal state, CPEB3 binds to target mRNA in the brain to repress translation [55]. However ample evidence has shown that CPEB3 can be converted into an activating state by post-translational modification that prompts an aggregated state, likely regulated by the N-terminus domain [74]. Regulatory mechanisms that facilitate these functional states include small ubiquitin-like modifier (or SUMOylation) which acts as an inhibitory constraint for CPEB3 aggregation [75] (Figure 2). In hippocampal neurons for example, basal SUMOylation was shown to maintain CPEB3 in its monomeric and repressive state. Indirectly, the glutamatergic agonist glycine increases CPEB3 and decreases SUMOylated CPEB3 [75]. This phenomenon was confirmed in vivo during fear conditioning learning. Indeed, the presence of SUMO at the CPEB3 N terminus prevents the aggregation of CPEB3 and the translation of the target mRNAs of CPEB3 in hippocampal neurons following glycine stimulation [75]. Interestingly, CPEB3 overexpression in neurons reduced translation of SUMO-2 mRNA in a CPE-dependent manner, suggesting a negative feedback regulatory loop between CPEB3 and SUMO-2, likely to limit the aggregation of CPEB3 [75]. Further, studies in yeast have suggested that stimulation-induced aggregation of CPEB3 and activation of downstream targets like GluA2 and actin are highly dependent on the PRD1 domain, supporting the importance of prion-like formation of CPEB3 to its regulatory function in neurons [73].

### CPEB3 Ubiquitination

On the other hand, some known activators of CPEB3, such as the E3 ubiquitin ligase Neuralized 1 (Neurl1), interact with CPEB3 in hippocampal neurons to monoubiquitinate and activate the protein through interaction with the N-terminal prion-like domain [76]. This mechanism leads to increased production of GluA1 and GluA2 and dendritic growth and is supported by the co-localization of mono-ubiquitinated CPEB3 and GluA1/2 along apical dendrites of adult Cornu Ammonis 1 (CA1) neurons in Neurl1-overexpressing mice. Interestingly, when CPEB3 was overexpressed, GluA1 and GluA2 were reduced, indicating that Neurl1 ubiquitination is required to activate the CPEB3-dependent polyadenylation and translation of GluA1 and GluA2 (Figure 3).

### Voltage-gated protein kinase interaction

The bilateral regulation of CPEB3 from repressor to activator is heavily dependent on the modified state of CPEB3, which can be triggered by signal-mediated activity, such as ion influx from voltage-gated channels (i.e. stimulation-dependent). On another regulatory plane, graded dendritic depolarization elevates CPEB3 protein at proximal dendrites, influencing the synthesis of PRPs like AMRAR subunits and consequently, shaping the synaptic gradient of excitatory receptors. For example, action potentials (APs) regulate CPEB3 expression by activating protein kinase C (PKC) via Ca^2+^ entry through voltage-gated Ca^2+^ channels. Once PKC is activated, it is translocated to the plasma membrane it mediates the post-synaptic activity dependent regulation of CPEB3 as demonstrated by PKC inhibitors [77]. Disruption of CPEB3-GluA2 mRNA interaction increases synaptic GluA2 expression at proximal synapses, indicating that CPEB3-GluA2 mRNA interactions may be responsible for the dendritic GluA2 gradient that fine-tunes the neuronal response to incoming signals. Indeed, inhibition of somatic AP firing with tetrodotoxin reduced the expression of CPEB3 and increased synaptic GluA2 AMPARs [77]. Thus, the activity-induced expression of CPEB3 suggests a cell-autonomous mechanism where sustained postsynaptic firing drives graded local proteins synthesis, consequently directing the spatial organization of synaptic AMPARs [77].

### Nuclear translocation

In neurons, NMDA activation promotes accumulation of CPEB3 in the nucleus. The nuclear translocation of CPEB3 is driven by the karyopherins importin 5 (IPO5) and chromosomal maintenance 1 (CRM1, also known as exportin 1) through binding of the ribonucleoside-diphosphate reductase large subunit (RRM1) domain of CPEB3. IPO5 binding is regulated by RanGTP and RanGDP. After NMDA stimulation, RanGDP is elevated in tandem with IPO5-mediated nuclear import of CPEB3 [78]. Preferential localization of nuclear CPEB3 is associated with Stat5b binding and the downregulation of Stat5b-dependent transcription. As previously mentioned, downregulation of the Stat5b target EGFR has been linked with diminished LTP, spatial learning and memory performance, indicating another mechanism by which CPEB3 influences synaptic plasticity and regulation of long term memory [26].

### Protein kinase A phosphorylation

Protein kinase A (PKA) phosphorylation of CPEB3 has also been observed downstream of NMDAR activation [79]. PKA phosphorylation likely occurs in serine residues S419 and S420 of the CPEB3a isoform, as no phosphorylation was induced in S419A/S420A double mutant peptides [80]. Interestingly, calcium 2+/calmodulin-dependent kinase II alpha (CaMKIIa) phosphorylation occurs on the same residues of CPEB3, located within exon 7 of the CPEB3 gene (B-region) which harbors the kinase recognition site. These kinases are likely mediators of sustainable alterations occurring on CPEB3 for synaptic plasticity events such as long term potentiation [79]. Indeed, induction of epileptic seizures in mice skews in favor of the production of B-region containing CPEB3 splice variants, likely to increase the ability of CPEB3 aggregates to become activated by phosphorylation [80]. Further experiments showed that the Thr286 auto-phosphorylation signal of CAMKIIa [63,64] is increased by NMDA-stimulation in CPEB3 KO neurons [81, 82]. Similarly, GluA1Ser831 phosphorylation (by CAMKIIa) [64.65] was also increased in [82, 83], effects which were both rescued by ectopic expression of CPEB3, indicating that the translational up-regulation of NMDA receptor (NMDAR) and PSD95 proteins is caused by the loss of CPEB3, directly accounting for impaired c-LTD (Figure 3).

### Stimulation-dependent polyadenylation

Finally, stimuli-dependent increases in AMPA subunits have been experimentally shown to be regulated by CPEB3-driven increases in translation of the targets through polyadenylation, suggesting a functional switch of the RNA-binding protein occurs in the context of synaptic stimulation [55]. However, another group found that in transfected neurons, the functional reversal of repressive CPEB3 (in response to NMDA treatment) occurs independent of cleavage and polyadenylation specificity factor (CPSF) or AAUAAA hexanucleotide interaction-two critical components of polyadenylation [39]. Discrepancies in these two studies may come down to differences in the model system, the experimental delivery of synaptic stimulation, or the methodology used to achieve CPEB3 downregulation.

### Stimulation-independent regulation of CPEB3

### P-bodies

Regarding the basal repressive role of CPEB3, recent evidence has found that CPEB3 is localized to membrane-less cytoplasmic P-bodies, subcellular compartments that are enriched in translationally repressed mRNA. After stimulation, CPEB3 is recruited into polysomes, thus promoting the translation of its target mRNAs. In HeLa cells, GFP-Ago2, CPEB3-DsRed, and HA-GW182 were co-localized in the cytoplasmic puncta identical to the distribution of P-bodies. Further, co-IP experiments showed that CPEB3 interacts with GW182 but only in cross-linked samples did it interact with argonaute 2 protein (Ago2). The binding of CPEB3 to P-body proteins was found to occur through the RRM1 domain as deletion of this element disrupts the co-localization of CPEB3 with HA-Ago2 and HA-GW182 [72]. Interestingly, localization of CPEB3 to P-bodies is driven by SUMOylation as demonstrated by SUMOylation inhibitor (Figure 2). In contrast, co-transfection of CPEB3 with SUMO promotes phase separation of CPEB3 *in vitro* and modulates the co-localization of CPEB3 with the P-body protein mRNA de-capping enzyme 1 (Dcp1).

### Basal state polyadenylation

CPEB3, like CPEB2 and CPEB4 contain cytoplasmic polyadenylation element (CPE) binding regions that are responsible for RNA binding and translational control at the CPEs of target mRNA. Consequently, the length of the polyA tails of target mRNA are correlated with the translational activity of the mRNAs [84], with translationally dormant mRNAs having shorter tails compared to active mRNA. Through this mechanism, CPEBs regulate polyadenylation of RNA-binding proteins to influence diverse biological processes from germ-cell development, cell division, to synaptic plasticity and learning and memory [85]. Interestingly, CPEB3 does not require the polyadenylation proteins cleavage and polyadenylation specificity factor (CPSF) nor the hexanucleotide AAUAAA, suggesting polyadenylation-independent translational activation [39]. On the other hand, the cell cycle regulator Tob, which directly binds to CPEB3 through the carboxyl-terminal RNA binding domain, has been shown to recruit the Caf1 de-adenylase to form a tertiary complex, accelerating the de-adenylation and decay of target mRNA as demonstrated in COS-7 cells (Figure 3) [86]. This interaction has been validated by reporter assay appended with the 3’ untranslated region (UTR) of GluA2, a known CPEB3 target, where a 10% protein reduction and 40% mRNA reduction was observed compared to a control. Interestingly, Tob has been previously implicated in the regulation of learning and memory [87, 88].

### eEF2 regulation

Another study proposed that CPEB3 regulation of GluA2 subunits may occur through the eukaryotic elongation factor 2 (eEF2), a translation factor essential for the translocation of the ribosome by GTP hydrolysis and a key regulator of the elongation step of translation [89]. Based on yeast two-hybrid screen and co-immunoprecipitation, CPEB2 was shown to directly interact with eEF2. Upon CPEB2 binding with eukaryotic elongation factor 2 (eEF2), ribosome activated GTP hydrolysis was diminished, leading to the repression of target RNA translation at elongation as demonstrated by reporter RNA. Since CPEB3 shares a 95% sequence identity with CPEB2 in the C-terminal RNA binding domain, it is plausible that eEF2-related interaction are also at work for the curation of CPEB3 downregulation of targets like NMDAR and PSD95 in its basal state [90]. However, in another study, experiments with a selective eEF2 kinase inhibitor showed neither a reduction in CPEB3 nor an increase in GluA2 [89] were observed, calling into question the role of eEF2 in the basal regulation of CPEB3.

## CONCLUSIONS AND PERSPECTIVES

Rapid, responsive local protein synthesis is the apex of activity related synaptic plasticity. For functions like long-term memory and learning, self-perpetuating mechanisms for the recurring generation of plasticity related proteins is the second piece of the puzzle. The discovery of the prion-like CPEB3 local protein synthesis regulator provided a very attractive answer to the long-elusive question of how local synaptic modulation is both achieved and maintained. Years of elegant research spanning multiple organisms and model systems have provided crucial insight into how precisely CPEB3 is activated/repressed, its downstream targets and the structural properties that allow such interactions, among other things.

While great strides have been made in the understanding of the self-perpetuating and aggregational nature of CPEB3, it is quite clear that more precise and tightly regulated controls are at work to maintain this and similar RNA binding proteins from running rampant in the synapse and taking a pathological turn. Thus, it is imperative to continue to endeavor to identify and characterize the factors that interact with CPEB3 to control the propagation of its prion-like state. Further, it is unlikely that CPEBs work in singular fashion, therefore, an improved understanding of the complimentary RNA binding proteins at work during neural modulation is critical to build a more accurate snapshot of real life synaptic plasticity. In a similar vein, modulation of RNA binding proteins like CPEB3 likely do not happen in a vacuum, meaning, glutamatergic stimulation likely triggers a myriad of regulatory events such as the activation of kinases, proteases, and karyopherins (among others) to act collectively upon CPEB3 at any given time in response to synaptic activation. What are the thresholds for activation and recruitment of these modulators to CPEB3 and which takes precedent after an excitatory event? These are just some of the questions that beckon further investigation. Finally, it remains important to continue characterizing both CPEB3 mRNA targets and the mechanisms and thresholds for bilateral regulation of these through CPEB3.
